# Recent advances in pancreatic disease

**Published:** 2015

**Authors:** David Al Dulaimi

**Affiliations:** *Department of Gastroenterology, Alexandra Hospital, Redditch, UK*

*Chatterjee S,* *et al.* ***Autoimmune pancreatitis diagnosis, management and longterm follow-up.*** *J Gastrointestin Liver Dis 2014;23(2):179-85*

The diagnosis and management of autoimmune pancreatitis (AIP) is challenging in certain patient groups. This retrospective study evaluated the diagnosis and management of AIP in 22 patients (95% male, mean age 64.8 y (range 43-84 y)) diagnosed at the Freeman Hospital, England, between 2005 and 2013. Type I (n=21) AIP was more common than type II AIP (n=1). Patients presented most frequently with abnormal liver function tests (LFTs) without jaundice (77%), weight loss (41%) and acute abdominal pain without hyperamylasemia (27%). Pancreatic exocrine insufficiency was identified in 41% and a further 23% of patients were diabetic at presentation. Total immunoglobulin IgG, IgG4 and Ca19-9 were elevated in 45%, 64% and 73% of patients respectively. Extrapancreatic involvement was prominent (68% of patients), most commonly bile duct strictures (64%), gall bladder and peripancreatic infiltration (9%) and retroperitoneal fibrosis (9%). All patients underwent a pancreatic protocol CT which most commonly identified pancreatic head enlargement (50%) and thickening/stricturing of the extrapancreatic (50%) and intrahepatic bile duct dilataion (45%). 

Corticosteroid therapy was initiated in 82% of patients. 23% relapsed and were treated with azathioprine. In all cases symptom alleviation was achieved following two weeks of treatment and Ca19-9 normalised. LFTs normalised in 12 patients by the fourth week of treatment. Four patients had sub-optimal response to immunosuppression with one death due to end stage liver failure. Overall the diagnosis and management of AIP is challenging and may be best achieved with a multi-disciplinary team. 

*Hadizadeh M, Padashi M, Mohammad Alizadeh AH, Zali MR.* ***Clinical, laboratory biomarkers and imaging findings of pancreatic adenocarcinoma in Iran.*** *Asian Pac J Cancer Prev 2014;15(10):4349-52.*

A clear understanding of the epidemiology of pancreatic cancer in Iran is important for early diagnosis. This retrospective study evaluated the clinical presentation, investigation and management of 131 patients with histologically-confirmed pancreatic adenocarcinoma (61.1% male, mean age 63 ± 3.4 y) admitted to Taleghani Hospital, Tehran, Iran from 2010 to 2013. Several risk factors for pancreatic adenocarcinoma were identified including diabetes (27.5%), smoking or alcohol history (21.4% and 3.9% respectively) and cholelithiasis (18.3%). Patients most commonly presented with anorexia (67.1%), weight loss (60.3%), abdominal pain (58.8%) and jaundice (55%). An elevated serum Ca 19-9 (>50 UmL^-1^) was detected in 81% of cases. Of note, 90.1% of cases were stage III or greater at diagnosis with tumour most frequently involving the pancreatic head (71.8%). Pancreatic duct dilatation was prominent (88% of patients) and 45 patients underwent ERCP with metallic or plastic stent insertion in 17 and 22 patients respectively. Percutaneous transhepatic cholangiography was completed in 6 patients. 

Surgical treatment with Whipple procedure was performed in 10 patients. 29 patients received chemotherapy, reflecting the advanced cancer stage at diagnosis. Clinicians should adopt a high index of suspicion for pancreatic cancer, an important differential diagnosis in patients with non-specific gastrointestinal symptoms. Aggressive health promotion is needed to modify risk factors and encourage early presentation.

*Wang Y, Zhang FC, Wang YJ.* *Helicobacter pylori* ***and pancreatic cancer risk: a meta-analysis based on 2,049 cases and 2,861 controls.*** *Asian Pac J Cancer Prev 2014;15(11):4449-54.*

*Helicobacter Pylori* (*H. Pylori*) infection is an established risk factor for many gastrointestinal cancers however an association with pancreatic cancer is unclear. This meta-analysis of 9 studies including 2049 cases and 2861 controls investigated the relationship between *H. Pylori *infection and pancreatic cancer in adults. There was no significant association between *H. Pylori* infection and pancreatic cancer when all cases were analysed or on sub-analysis of cases from Western population studies (7 of 9). Interestingly in the Eastern study populations (1 China, 1 Japan) *H. Pylori* infection was associated with decreased pancreatic cancer risk (odds ratio (OR) 0.62, 95% confidence interval (CI) 0.49-0.76). Similarly infection with *H. Pylori* expressing cytotoxin-associated gene A (Cag A) decreased the risk of pancreatic cancer in patients from Eastern countries only (OR 0.66, 95%CI 0.52-0.80). These findings may reflect variation in the predominant strain of *H. Pylori *in Western and Eastern populations. The authors postulate that *H. Pylori* infection may be cancer protective by modulating appetite leading to reduced obesity. These results should be validated by further large, standardised studies in Eastern and Western populations.

*Hocke M, Cui XW, Domagk D, Ignee A, Dietrich CF.* ***Pancreatic cystic lesions: The value of contrast-enhanced endoscopic ultrasound to influence the clinical pathway.*** *Endosc Ultrasound 2014:3(2)123-30.*

The lack of clear clinical criteria to discriminate malignant from benign cystic pancreatic lesions causes management uncertainty. This retrospective study of 125 patients with cystic lesions of the pancreas led to creation of a two-step diagnostic pathway using contrast-enhanced endoscopic ultrasound and endoscopic fine needle aspiration. Firstly, contrast-enhanced endoscopic ultrasound should be used to evaluate for signs of cystic neoplasia in the pancreas including cystic wall diameter >3cm, septae, nodules within the cyst, pancreatic duct dilatation and cystic wall vascularisation. If any of these features are identified, endoscopic fine-needle aspiration of the lesion with prophylactic antibiotics is indicated to evaluate malignant potential. Lesions with mucous liquid, carcinoembryonic antigen (CEA) level >400 or which meet cytological tumour criteria should be considered for surgical intervention. Follow-up is advised for patients with serous liquid, CEA <400 and cytological normal cells. Although clinically useful, it is important for future studies to evaluate the effectiveness of this proposed diagnostic pathway.

*Okasha HH et al.* ***Endoscopic ultrasound-guided fine needle aspiration versus percutaneous ultrasound-guided fine needle aspiration in diagnosis of focal pancreatic masses.*** *Endosc Ultrasound 2013:2(4)190-193.*

Achieving an early histopathological diagnosis of pancreatic cancer is important to target treatment and reduce morbidity and mortality. This multi-centre prospective study of 197 consecutive patients (77% male) with pancreatic head masses from 2008 to 2013 compared the diagnostic accuracy of endoscopic ultrasound-guided fine needle aspiration (EUS-FNA) (n=72) with percutaneous ultrasound-guided fine needle aspiration (US-FNA) (n=125). A sensitivity of 84%, specificity of 100%, positive predictive value (PPV) of 100%, negative predictive value (NPV) of 73% and overall accuracy of 89% was demonstrated with EUS-FNA compared to a sensitivity of 86%, specificity of 90%, PPV of 95%, NPV of 76% and overall accuracy of 87% with US-FNA. A complication rate of 1.4% and 5.6% was observed with EUS-FNA and US-FNA respectively. Notably three patients who underwent US-FNA had procedure related tumour seeding. Endoscopic ultrasound allowed aspiration of smaller lesions compared with percutaneous ultrasound (9mm and 20mm respectively). Despite similar diagnostic accuracies the authors conclude EUS-FNA is preferable to US-FNA given the lower complication rate and smaller size of accessible lesions. Diagnostic accuracy may be enhanced by adopting a three pass approach and on-site cytopathology facilities.

*Tenner S, Baillie J, DeWitt J, Vege SS* *.* ***American College of Gastroenterology guideline: management of acute pancreatitis.*** *Am J Gastroenterol 2013:108(9)1400-15.*

An evidence-based approach to the diagnosis, investigation and management of acute pancreatitis (AP) is provided by this American College of Gastroenterology guideline constructed following a MEDLINE search of clinical trials, reviews, guidelines and meta-analysis related to AP between 1966 and 2012. In general the diagnosis of AP requires two out of three features: congruent abdominal pain history, serum amylase/lipase greater than three times the upper limit of normal and characteristic imaging findings. A transabdominal ultrasound scan should be performed in all AP patients to investigate for gallstones. In the absence of gallstones or a significant alcohol history serum triglycerides should be measured. Cases of idiopathic AP should be referred to a specialist centre and genetic testing should be considered in young patients (<30 y) with unclear aetiology and a family history of pancreatic disease. The role of contrast-enhanced CT and MRI is limited.

In the absence of significant comorbidities early aggressive intravenous fluid resuscitation, preferably with lactate Ringer's solution, should be provided to prevent complications such as pancreatic necrosis. The presence of organ failure signifies moderate-severe AP and a need for management in an intermediary/intensive care unit. ERCP should be completed within 24 h of admission in patients with AP and acute cholangitis but early ERCP is not generally required in gallstone pancreatitis without ongoing biliary obstruction. ERCP with guidewire cannulation is preferable to radiocontrast since pancreatic hydrostatic injury is avoided. EUS or MRCP should be used to screen for choledocholithiasis if highly suspected in the absence of cholangitis and/or jaundice. Pancreatic duct stents and/or post procedure rectal NSAID suppositories should be used to lower the risk of severe post-ERCP pancreatitis in high-risk patients. 

The prevention and early treatment of infected pancreatic necrosis is important to reduce mortality however prophylaxis with antibiotics or anti-fungal agents is not recommended in cases of severe AP or sterile pancreatic necrosis. Material from infected pancreatic necrosis should be sampled by CT guided fine needle aspiration or minimally invasive drainage for microscopy and culture to guide treatment. Contrary to historical practice, enteral feeding is indicated immediately in AP without nausea or abdominal pain and may help to reduce infectious complications. Evidence suggests a low fat solid diet is as safe as a clear liquid diet and nasogastric and nasojejunal feeding offer similar efficacy. Cholecystectomy should be performed prior to discharge in cases of mild gallstone pancreatitis to prevent recurrence but should be delayed until fluid collection stabilises in necrotising biliary AP. Surgery is not required in cases of asymptomatic pancreatic pseudocysts or necrosis. In stable patients with infected necrosis drainage should be delayed by four weeks to allow necrosis liquefaction and walling-off.

**Papers were prepared by:**

**Drs Luke Materacki **and** Ishfaq Ahmad, **Worcestershire Acute Hospitals NHS Trust, UK

